# Optimized Alkaline Extraction and Functional Characterization of Carrageenan from *Eucheuma perplexum* Using Response Surface Methodology

**DOI:** 10.3390/foods14203496

**Published:** 2025-10-14

**Authors:** Irene P. Daet, Tai-Yuan Chen, Sharon N. Nuñal, Jose P. Peralta, Rhoda Mae C. Simora, Meng Chou Lee, Jui-Sheng Chang, Rex Ferdinand M. Traifalgar

**Affiliations:** 1 Institute of Fish Processing Technology, College of Fisheries and Ocean Sciences, University of the Philippines Visayas, Miagao 5023, Iloilo, Philippines; daet.irene@parsu.edu.ph (I.P.D.); f153mentor@yahoo.com (J.P.P.); rcsimora@up.edu.ph (R.M.C.S.); 2 College of Fisheries and Marine Science (Sagñay Campus), Partido State University, Nato, Sagñay 4421, Camarines Sur, Philippines; 3 Department of Food Science, Institute of Food Safety and Risk Management, National Taiwan Ocean University, Keelung 20224, Taiwan; 4 Department of Aquaculture, National Taiwan Ocean University, Keelung 20224, Taiwan; mengchoulee@email.ntou.edu.tw; 5 Center of Excellence for the Oceans, Department of Aquaculture, National Taiwan Ocean University, Keelung 20224, Taiwan; jschang@email.ntou.edu.tw; 6 Institute of Aquaculture, College of Fisheries and Ocean Sciences, University of the Philippines Visayas, Miagao 5023, Iloilo, Philippines; rmtraifalgar@up.edu.ph

**Keywords:** carrageenan, *Eucheuma perplexum*, seaweed hydrocolloids, alkaline extraction, response surface methodology, emulsifying activity

## Abstract

Seaweeds are increasingly valued in the food industry for their bioactive compounds, mainly hydrocolloids like carrageenan. This study investigates *E. perplexum*, a red seaweed, as a unique and sustainable source of carrageenan with promising functional properties. Using response surface methodology (RSM), the extraction process was optimized through alkaline extraction, identifying optimal conditions of 85 °C for 3 h with 2.58 M KOH, which yielded 77.10% carrageenan. The extracted carrageenan exhibited strong emulsifying activity (71.53 ± 2.41) and color properties comparable to commercial carrageenan, highlighting its viability for food applications. Chemical evaluation revealed a higher sulfate content (8.45 ± 0.16) and slightly reduced carbohydrate levels, which may influence its gelling and stabilizing abilities. Structural examination through ATR-FTIR spectroscopy corroborates the presence of key functional groups, including sulfate esters and galactose derivatives, inferring molecular integrity. These results emphasize the importance of RSM in optimizing extraction and underscore the ability of *E. perplexum* as a promising source of the derived carrageenan, which is a high-performance additive in food systems. Further research on purification, functional enhancement, and safety assessment is recommended to facilitate its integration into commercial food systems.

## 1. Introduction

*Eucheuma perplexum*, a red seaweed belonging to the family Solieriaceae under the order Gigartinales, has attracted attention as a promising source of carrageenan—a natural hydrocolloid widely utilized as a thickener and stabilizer in food systems. Carrageenans are linear polysaccharides composed of D-galactose and 3,6-anhydro-D-galactose units. Owing to their distinctive gelling, thickening, stabilizing, and emulsifying properties, carrageenans are highly valued across the food, cosmetic, and pharmaceutical industries [1].

Globally, carrageenan production relies predominantly on *Eucheuma* and *Kappaphycus* species, which together account for more than 90% of the raw material supply [2]. In 2019, the total harvest of carrageenan-producing seaweeds reached approximately at 11.7 million tonnes (wet weight), with Southeast Asian nations leading the supply chain [3]. Key contributors include China, Indonesia, the Philippines, Malaysia, and Tanzania, all of which show vital roles in the global market [4].

The international carrageenan industry was assessed at around USD 802 million in 2022 and is projected to reach USD 1.1 billion by 2030, driven by increasing demand for processed foods, dairy products, and pharmaceutical uses [5]. The Philippines remains central to this industry, contributing nearly 60% of the global dried seaweed supply and generating USD 185 million in carrageenan exports in 2018 (28,018 MT) [6,7]. Carrageenan accounts for roughly 94% of the country’s total seaweed export value, highlighting its importance to aquaculture and coastal livelihoods [7].

While *Eucheuma denticulatum* and *Kappaphycus alvarezii* dominate commercial cultivation, *E. perplexum* has received limited attention. Nevertheless, its biochemical resemblance to other eucheumatoids suggests considerable potential as an alternative carrageenan source. Investigating this underutilized species could diversify the raw material base and strengthen the resilience of seaweed farming communities. Hence, *E. perplexum* represents a promising and sustainable carrageenan resource with implications for both industrial expansion and socio-economic development.

Carrageenan extraction typically involves hot aqueous or alkaline treatments using agents such as potassium hydroxide (KOH) or sodium hydroxide (NaOH). The yield and quality of the final product are highly dependent on processing parameters [8]. Variables such as seaweed-to-water ratio, temperature, extraction time, and alkali concentration significantly influence dissolution behavior, yield, and gel properties [9,10]. For instance, higher KOH concentrations may improve gel strength but can also reduce yield and viscosity [11,12]. Optimizing these factors is therefore critical to maximize both carrageenan quality and extraction efficiency.

To achieve this, statistical approaches such as Response Surface Methodology have been widely applied in polysaccharide research. RSM has proven effective in optimizing extraction conditions, preserving molecular weight, and enhancing functional attributes in both seaweed- and plant-based polysaccharides [13,14,15,16,17]. In carrageenan studies, RSM has successfully optimized key factors such as alkali concentration, temperature, and extraction time, leading to the production of semi-refined or refined carrageenan with desirable gel characteristics [12,18,19,20].

Building on these findings, the present study employed RSM with a Central Composite Design (CCD) to optimize the alkaline extraction of carrageenan from *E. perplexum* for maximum yield. Furthermore, the physicochemical and functional characteristics of the optimized extract, including emulsifying activity, color metrics, sulfate and carbohydrate composition, viscosity, and structural features analyzed by ATR-FTIR spectroscopy, were comprehensively evaluated to asess its potential as a high-value industrial hydrocolloid.

## 2. Materials and Methods

### 2.1. Collection of Raw Material, Preparation, and Drying of E. perplexum

Fresh specimens of *E. perplexum,* previously identified by Liao et al. [21], were collected from cultivated stocks maintained at the Algae Resources Laboratory, Center of Excellence for the Oceans, National Taiwan Ocean University, Taiwan, from September to December 2022. The harvested seaweed was immediately packed in polyethylene bags, placed in styrofoam containers, and transported to our laboratory within 10 min. Upon arrival, the samples were immediately thoroughly rinsed with tap water to eliminate epiphytes, sand, silt, residual macroalgae, and excess salt. The cleaned samples were then stored at −20 °C to preserve their biochemical integrity until further processing.

To determine the optimal drying parameters, preliminary experiments were conducted by testing different combinations of temperature and time, using moisture content as the primary indicator. Prior to drying, frozen samples were thawed at 4 °C for 12 h, washed with distilled water, cut into smaller pieces, and subjected to hot air drying under selected conditions. A drying protocol of 70 °C for 24 h was ultimately adopted, as it ensured sufficient dehydration while preserving the structural integrity of the biomass. The resulting dried biomass was milled into a fine powder, packaged in airtight containers, and stored at −20 °C for subsequent analytical procedures.

### 2.2. Determination of Dried E. perplexum, Water Activity, and Moisture Content After Drying

The yield (%) of dried *E. perplexum* was determined to quantify the actual biomass available for subsequent carrageenan extraction. Since fresh seaweed contains a high proportion of water, calculating the dried yield provides a standardized measure of dry matter, minimizing variability caused by differences in initial moisture content. The dried yield was calculated as the ratio of the dry weight obtained after drying to the initial fresh weight of the seaweed, multiplied by 100, as expressed in Equation (1):

(1) $$Yield\;\left(\%\right)=\frac{Dry\;weight\;of\;sample}{Fresh\;weight\;of\;seaweed}\times 100$$

Moisture content was assessed through a conventional gravimetric method, wherein samples were dried to a constant weight in a hot-air oven maintained at 105 °C [22]. Water activity (Aw) was evaluated at ambient temperature using a properly calibrated AquaLab CX-2 m (Smartec Scientific Corp., New Taipei City, Taiwan).

### 2.3. Optimization of Carrageenan Extraction from E. perplexum

Carrageenan extraction from *E. perplexum* was optimized using Response Surface Methodology with a Central Composite Design to systematically evaluate three key independent variables: extraction temperature (°C), extraction duration (hours), and potassium hydroxide concentration (M), examining their impact on carrageenan yield. These parameters were selected based on a comprehensive review of existing literature, highlighting their critical roles in yield and quality of extracted carrageenan [18,23,24]. Specifically, temperature influences polysaccharide solubility, KOH concentration affects alkaline extraction efficiency and sulfate group stability, and extraction time determines the release of carrageenan from the biomass. The CCD framework within RSM was chosen for its capacity to efficiently estimate linear, interaction, and quadratic effects with a limited number of experimental runs. Variable levels were determined through our preliminary trials and literature to ensure coverage of conditions conducive to optimal extraction without compromising product integrity. These parameters were incorporated into the experimental matrix (Table 1) to facilitate a structured investigation of their individual and combined effects. The experimental design consisted of 20 runs; each was performed in triplicate to ensure statistical reliability. Although 110.22 °C is not practically achievable in the actual experiment (since the process was limited to 100 °C), the model deliberately explores values beyond the experimental range to establish the axial (±α) points. These points are essential for capturing curvature and interaction effects among the three independent variables. They enable more accurate fitting of the quadratic model and ensure rotatability. While the experiment was limited to 100 °C, keeping these design points in the table is necessary to preserve the model’s integrity and the statistical analysis (Table 2). Randomization of trials was implemented to mitigate the influence of uncontrolled experimental variability. Design-Expert software (Version 11; Stat-Ease Inc., Minneapolis, MN, USA) was employed to develop the experimental matrix, conduct statistical modeling and analysis, and visualize the interaction effects through three-dimensional response surface plots. The software produced second-order polynomial models, which were subsequently validated under the predicted optimal conditions. The statistical relevance of the model coefficients was evaluated through analysis of variance (ANOVA), whereas the predictive accuracy and goodness-of-fit of the model were quantified using the coefficient of determination (R^2^) and its adjusted form (R^2^adj). The robustness and predictive accuracy of the models were further confirmed through the Fisher F-test, based on the ratio of mean squares.

#### Extraction and Recovery of Crude Carrageenan

Before the extraction process, a preliminary optimization study was performed in the laboratory to determine the ideal seaweed-to-water ratio using RSM-CCD. Ratios ranging from 1:25 to 1:50 g/mL were evaluated, and the analysis revealed that a ratio of 1:45 g/mL yielded the highest carrageenan output. Consequently, this ratio was selected for use in the present investigation. Interestingly, the optimized ratio of 1:45 g/mL aligns with the findings of Hasizah et al. [23] for *Eucheuma spinosum*, thereby reinforcing the validity of this parameter.

Carrageenan extraction was performed following the method of Torres et al. [18], with slight modifications. After extraction, phase separation was achieved by centrifugation at 4000 rpm for 10 min at 20 °C. Carrageenan was precipitated from the supernatant using 95% (*v*/*v*) ethanol at a 2:1 ratio (ethanol: supernatant, *v*/*v*), resulting in two fractions: a carrageenan-rich precipitate and a carrageenan-free liquid. The precipitate was collected by vacuum filtration and dried in a convection oven at 40 °C for 24 h. The dried material was designated as crude carrageenan and stored at −22 °C for further analyses.

### 2.4. Characterization of Carrageenan Extract

#### 2.4.1. Carrageenan Yield and Carbohydrate Content

The extraction yield (%) was calculated as the ratio of the mass of dry carrageenan powder obtained to the mass of the initial dried seaweed, following Firdayanti et al. [25]. Yield was determined using Equation (2):

(2) $$Yield\;\left(\%\right)\;\frac{\;Total\;mass\;of\;carrageenan\;extract\;\left(g\right)}{Mass\;of\;dried\;seaweed\;\left(g\right)}\times 100$$

The total carbohydrate content of the extracted carrageenan was determined according to the method of Deans et al. [26]. Commercial κ-carrageenan (Catalog No.22048, Sigma-Aldrich Co., St. Louis, MO, USA; CAS No. 11114-20-8) was used as a reference to compare the results obtained in this study.

#### 2.4.2. Emulsifying Properties

The emulsifying ability and stability of crude carrageenan (CCGN) were evaluated in comparison with commercial κ-carrageenan (KCGN), which served as the standard control, following the method of Zhang et al. [27], with slight modifications. Soybean oil was chosen as the oil phase and mixed with aqueous carrageenan solutions in equal volumes (10 mL oil + 10 mL solution), resulting in a 50% oil fraction. Emulsions were prepared in 50 mL tubes using a high-speed homogenizer at 8000 rpm for 2 min. No surfactants or salts were added; carrageenan was the only emulsifier used. The initial height of the oil-solution mixture (*H*_0_, cm) was recorded before homogenization. After emulsification, samples were left to stand for 10 min, and the height of the emulsion layer (*H*_1_, cm) was measured. Additional measurements of the emulsion layer height (*H*_2_, cm) were taken at intervals of 30 min, 1 h, 1.5 h, 2 h, 2.5 h, and 3 h. All tests were performed in triplicate. Emulsifying capacity and emulsion stability were determined using the following equations:

(3) $$Emulsifying\;ability\;\left(\%\right)=\;\frac{H_{1}}{H_{0}}\;\times \;100$$

(4) $$Emulsifying\;stability\;\left(\%\right)=\frac{H_{2}}{H_{1}}\times 100$$

where *H*_0_ is the initial height of the mixture, *H*_1_ is the height of the emulsion layer after 10 min, and *H*_2_ is the height of the emulsion layer at subsequent time intervals.

#### 2.4.3. Color Properties

The color parameters (L*, a*, and b*) of the extracted carrageenan (CCGN) were measured using a Tokyo Denshoku TC-1800MK-II Colorimeter (Shinjuku, Tokyo, Japan), with commercial κ-carrageenan (KCGN) serving as the reference control. The chromaticity coordinates (a* and b*) were then used to compute the chroma (C*) and hue angle (H°). The overall color difference (ΔE) between samples was calculated using Equation (5) following the standard formula of McGuire [28].

(5) $$\Delta E=\sqrt{(\Delta {L)}^{2}+(\Delta {a)}^{2}+(\Delta {b)}^{2}}$$

where Δ*L* is the difference in lightness between the sample and reference, Δ*a* is the difference in the red–green coordinate between the sample and reference, and Δ*b* is the difference in the yellow–blue coordinate between the sample and reference.

#### 2.4.4. Sulfate Content

The sulfate content of the carrageenan was determined using the method of sulfate hydrolysis followed by precipitation of sulfate as barium sulfate [29]. A known amount of dried carrageenan sample (W_1_, g) was hydrolyzed by adding 50 mL of 1N HCl for 30 min. While boiling, 10 mL of 0.25M BaCl_2_ was added dropwise for 5 min. The mixture was then cooled at room temperature for 5 h to allow complete precipitation. The resulting BaSO_4_ precipitates were filtered using an ashless paper filter (Whatman No. 42, Cytiva, Marlborough, MA, USA) and then burned in a furnace for 1 h at 700 °C. The white ash was weighed (W_2_), and the sulfate content (%) was calculated using the equation:

(6) $$\%\;sulfate=\left(\frac{W_{2}}{W_{1}}\right)\times \;100\;\times \;0.4116\;$$

where *W*_1_ is initial dry weight of the carrageenan sample (g) and *W*_2_ is weight of the white ash (BaSO_4_) after incineration (g).

#### 2.4.5. Viscosity

The viscosity of crude carrageenan was measured using the method described by Distantina et al. [11] with slight modifications, wherein, viscosity of a dilute concentration (C) solution was measured. The sample was dissolved in distilled water by heating and then filtered through a paper filter before viscosity measurement. A capillary viscometer (TAMSON TMV40, Tamson Instruments B.V., Zoetermeer, The Netherlands) was used to measure the passage time of solution (t) and solvent (to) through the capillary at room temperature (24.5–27 °C).

#### 2.4.6. ATR-FTIR Analysis

The functional groups of the carrageenan samples were analyzed by attenuated total reflectance-Fourier transform infrared spectroscopy (ATR-FTIR) for a qualitative description. These measurements were evaluated using a Nicolet iS5 spectrometer (Thermo Fisher Scientific Inc., Waltham, MA, U.S.A)(Thermo iS5) from 400 to 4000 cm^−1^.

### 2.5. Statistical Analysis

Design-Expert software (Version 11, Stat-Ease, Inc., Minneapolis, MN, USA) was used to develop the experimental plan for Response Surface Methodology, perform regression analysis, estimate the coefficients of the regression equation, and conduct analysis of variance (ANOVA). All experimental data are presented as mean ± standard deviation (SD) of three replicates per sample. Differences between treatments were evaluated using ANOVA, and Duncan’s Multiple Range Test (DMRT) was applied to determine statistically significant differences at *p* < 0.05.

## 3. Results and Discussion

### 3.1. Optimization of Carrageenan Extraction

#### 3.1.1. Carrageenan Yield and Properties

Key factors influencing carrageenan production were evaluated during the optimization process using dried *E. perplexum* samples, rather than the fresh biomass, to better reflect conditions relevant for industrial applications. The fresh *E. perplexum* samples had a moisture content of 82.23%, which decreased to 27.66 ± 2.08% after drying at 70 °C. Similarly, the water activity decreased from 0.98 in the fresh samples to 0.42 after drying. This reduction in moisture and water activity not only facilitates handling and extraction but also indicates improved product quality and suggests an extended shelf-life for the dried seaweed. Additionally, optimization of the seaweed-to-water ratio, as detailed in Section “Extraction and Recovery of Crude Carrageenan” was critical in achieving maximum carrageenan yield.

The carrageenan yield obtained in this study ranged from 0 to 77.49% (Table 2). Variations in yield are commonly observed depending on the extraction methods and conditions. The yields obtained here are consistent with previously reported values, such as those from *E. spinosum* (29.6–65.77%) [14,23], *Mastocarpus stellatus* (40.3–60%) [30,31], and *K. alvarezii* (25.38–64.43%) [23,24]. These findings indicate that the experimental variables investigated significantly influenced carrageenan yield. Moreover, the high yield obtained from *E. perplexum* suggests that this species represents a viable alternative source of seaweed for carrageenan production.

The reduced quadratic regression model was evaluated using analysis of variance (ANOVA), which produced a Model F-value of 3.72, confirming the statistical significance of the model terms at a confidence level of *p* < 0.05 (Table 3). Notably, both the linear and quadratic components of KOH concentration (C and C^2^) exhibited significant effects on carrageenan recovery. The lack-of-fit F-value of 1.47 indicated no significant deviation between the model and the observed data, thereby confirming the model’s suitability for describing the experimental outcomes. The coefficient of determination (R^2^) was calculated at 0.60, demonstrating that 60% of the total variation in carrageenan yield could be explained by the combined effects of extraction temperature, time, and KOH concentration. This supports the suitability of the second-order polynomial model for describing the extraction process. In general, R^2^ reflects the proportion of total variance in the response variable accounted for by the model, with higher values denoting improved predictive accuracy [32,33].

#### 3.1.2. Effect of Extraction Conditions on the Yield of Carrageenan

**Figure 1:**
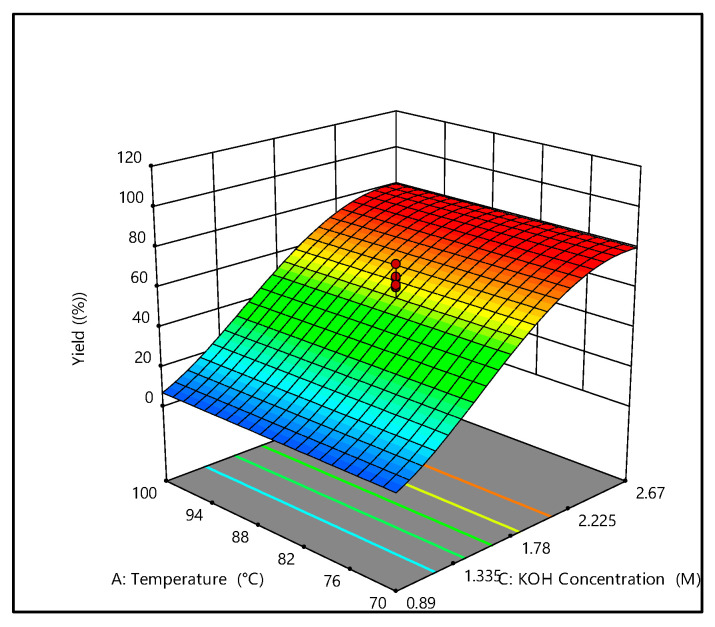
Three-dimensional graphic surface optimization of carrageenan yield of *E. perplexum* versus extraction temperature and KOH concentration.

Figure 1 illustrates the response surface and contour plots showing how extraction temperature and potassium hydroxide concentration jointly affect carrageenan yield from *E. perplexum*. The data indicate a strong dependence on KOH concentration, with higher levels significantly boosting extraction efficiency. Among the variables tested, KOH concentration proved to be the most influential, exhibiting a clear positive relationship with yield. This enhancement is likely due to the alkaline medium’s ability to break down the algal cell wall structure, thereby facilitating carrageenan release. Moreover, increased KOH concentrations may aid in converting precursor polysaccharides into κ-carrageenan by eliminating pyruvate residues and excess sulfate groups, further improving extraction outcomes [22,34,35,36,37].

At lower KOH concentrations (e.g., 0.89 M), carrageenan recovery dropped to zero, even when extraction time and temperature were increased (Table 2, Figure 1). Several trials (Runs 8, 9, and 17–19) resulted in no carrageenan yield, representing true zero values rather than experimental anomalies. These outcomes are attributed to insufficient alkalinity, which failed to disrupt the cell wall and release carrageenan. Under such conditions, polysaccharide solubilization was minimal, and elevated temperatures may have exacerbated degradation or hindered phase separation during precipitation. Additionally, poor filtration performance likely contributed to the low yield, as inadequate solubilization leads to higher solid residue retention. These findings emphasize the critical role of alkali concentration in carrageenan extraction and are consistent with previous studies that highlight the necessity of surpassing certain KOH and temperature thresholds for effective recovery [23,34].

Notably, Hasizah et al. [23] reported a 61.6% yield using only 0.4 M KOH with ohmic heating. This contrast highlights the impact of extraction methodology; unlike conventional heating, ohmic heating delivers rapid and uniform internal heating, along with electroporation effects that enhance cell permeability and mass transfer. These mechanisms enable efficient carrageenan recovery even at lower alkali concentrations. In comparison, the zero yields observed for *E. perplexum* under conventional heating suggest species-specific traits such as a more resilient or uniquely structured cell wall that demand stronger alkaline conditions for optimal extraction.

As carrageenan extraction from *E. perplexum* has not been previously documented, comparisons were made with other red seaweed species. For instance, *K. alvarezii* achieved a 76.7 ± 1.4% yield via ultrasound-assisted extraction with 8% KOH (≈1.42 M) [32], while conventional methods produced a 59.4% yield at 90 °C for 5 h [35]. In contrast, *E. cottonii* and *E. denticulatum* typically yield lower amounts (~20–33%) under standard alkaline conditions [38,39]. The relatively modest yield from *E. perplexum* under moderate KOH levels suggests either a more robust cell wall or a unique precursor composition, necessitating stronger alkaline conditions for optimal extraction. This reinforces the importance of tailoring extraction protocols to the biochemical characteristics of each seaweed species.

Extraction time and temperature also significantly influenced yield due to their impact on mass transfer dynamics [40]. Moderate temperature increases improved recovery by enhancing diffusion between the solvent and algal tissue [41]. However, excessive heat led to reduced yields, likely caused by polysaccharide breakdown, structural damage, and challenges in separating viscous filtrates from solid residues [12,35,42,43]. Similarly, while adequate extraction time ensured complete carrageenan solubilization, overly extended durations promoted depolymerization, diminishing yield, and potentially affecting product quality. Hence, the interaction between KOH concentration, temperature, and extraction time governs the balance between efficient carrageenan release and structural preservation. Optimal conditions were identified as 85 °C, 3 h, and 2.58 M KOH, yielding a predicted recovery of 77.10% (Figure 1). These parameters were validated through confirmatory experiments, which achieved an actual yield of 76.14%. This result is notably high compared to similar alkaline extraction studies [23,34,36], demonstrating the effectiveness of the optimized protocol for *E. perplexum*.

### 3.2. Characterization of Carrageenan

#### 3.2.1. Emulsifying Properties

**Figure 2:**
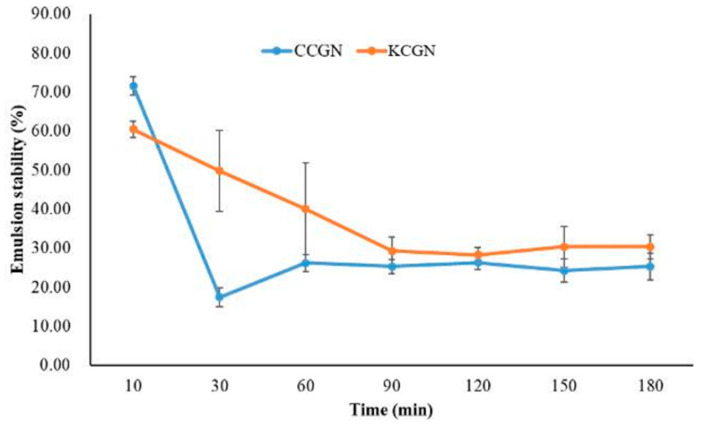
Emulsifying stability (ESI) and the ability of *E. perplexum.* CCGN stands for crude *E. perplexum* carrageenan, and KCGN for commercial carrageenan. Values are expressed as mean ± standard deviation (*n* = 3). Error bars represent variability among replicates.

The emulsifying performance of crude carrageenan was assessed through two key indices: emulsifying ability and emulsifying stability (ESI). Emulsifying ability reflects the carrageenan’s capacity to facilitate emulsion formation and stabilization, while ESI quantifies its effectiveness in maintaining emulsion integrity over time by resisting destabilizing phenomena such as coalescence, creaming, flocculation, and sedimentation [44]. In a soybean oil–water emulsion system containing 50% oil, prepared via high-speed homogenization (8000 rpm for 2 min) without the use of surfactants or salts, crude *E. perplexum* carrageenan (CCGN) showed higher emulsifying ability at the 10 min mark (71.53 ± 2.41) than commercial κ-carrageenan (KCGN) (60.42 ± 2.08). After 180 min at a concentration of 0.02%, CCGN maintained an emulsion stability of 25.25 ± 3.50, whereas KCGN exhibited a slightly higher value of 30.30 ± 0.03. The stability of emulsions formed by *E. perplexum* carrageenan is largely attributed to its hydrophilic–hydrophobic interactions, rather than simple partitioning between the aqueous and oil phases. Its hydrophobic regions are instrumental in anchoring lipid molecules, thereby enhancing the emulsification process [45]. The error bars shown in Figure 2 represent the standard deviation of replicate measurements, indicating variability in emulsifying performance between crude and commercial carrageenan. Overall, CCGN demonstrated strong emulsifying performance and successfully produced stable oil-in-water emulsions, as illustrated in Figure 2.

Importantly, this study is the first to report on carrageenan extraction and functionality from *E. perplexum*. While crude carrageenan exhibited superior short-term emulsifying ability, commercial κ-carrageenan outperformed in long-term stability. These findings suggest that *E. perplexum* carrageenan possesses unique biochemical features that may be harnessed for targeted applications, particularly in products requiring rapid emulsification, thereby positioning it as a novel and underexplored source of functional hydrocolloids for the food industry.

#### 3.2.2. Color Properties

The color characteristics of carrageenan extracted from *E. perplexum* are summarized in Table 4. Lightness (L*) values ranged from 84.70 in Run 10 to 94.90 in Run 1, indicating a general tendency toward the white spectrum (L = 100). Samples with L* values between 84.91 and 88.84 exhibited greater variability in brightness. These results are consistent with previously reported L* values for *C. canaliculatus*, which ranged from 91.18 to 91.34 and reflected a bias toward whiteness [46]. The observed increase in lightness may be attributed to improved penetration of the alkaline solution into the seaweed matrix, enhancing pigment removal during extraction. Regarding chromaticity, all samples displayed negative a* values, suggesting a shift toward green tones, and positive b* values, indicating yellow hues. Notably, the total color difference (ΔE) values for Runs 1 (10.86) and 14 (12.35) were significantly higher (*p* < 0.05) than those of other treatments, signifying more pronounced deviations in color. Commercial κ-carrageenan (Catalog No.22048, Sigma-Aldrich Co., St. Louis, MO, USA; CAS No. 11114-20-8) was used as a reference and exhibited a light cream appearance, which differed visibly from the experimental samples. These differences likely stem from variations in extraction protocols and the intrinsic properties of the seaweed species employed. Additionally, a clear trend was observed in which increasing extraction temperature led to a reduction in color intensity, as shown in Table 4. Overall, color evaluation serves as a critical quality indicator for carrageenan, reflecting both processing efficiency and product purity.

#### 3.2.3. Physiochemical Properties, Including Sulfate, Total Carbohydrate Contents, and Viscosity for Carrageenan Extraction from *E. perplexum*

The crude carrageenan extracted from *E. perplexum* exhibited a notably higher sulfate concentration (8.45 ± 0.16%) compared to the commercial counterpart (5.27 ± 0.16%). This finding aligns with the sulfate levels reported by Paz-Cedeño et al. [47], who documented values ranging from 8.1% to 13.8% in refined carrageenan. A downward trend in sulfate content with increasing KOH concentration has also been observed [11], suggesting that alkaline conditions may promote structural modifications such as cyclization and desulfation, thereby reducing sulfate levels. These variations may also reflect differences in seaweed species and extraction methodologies.

Sulfate groups play a pivotal role in defining carrageenan’s gelling capacity and functional performance. Their presence influences the polysaccharide’s behavior as a thickening, stabilizing, and gelling agent in various industrial applications. Carrageenan with moderate sulfate substitution tends to form stronger gels through ionic interactions, which can be tailored for specific food and pharmaceutical formulations. Additionally, sulfate content affects emulsifying properties: optimal substitution enhances molecular interactions with proteins and lipid droplets, thereby improving emulsion stability [48,49]. In contrast, excessive sulfate levels may hinder gel formation and emulsification due to increased electrostatic repulsion among charged groups.

Regarding carbohydrate composition, the crude extract contained 32.66% total carbohydrates, significantly lower than the commercial sample (53.47%) and previously reported values for *E. spinosum* (69.07–69.66%) [50]. These results underscore the importance of carbohydrate content as a marker of both functional quality and economic value. Thus, refining extraction conditions is essential to enhance carbohydrate retention and overall yield.

Viscosity measurements using a capillary viscometer (TAMSON TMV40, Tamson Instruments B.V., Zoetermeer, The Netherlands ) revealed an intrinsic viscosity of 16.90 ± 0.04 for the crude extract, substantially lower than that of commercial carrageenan (86.29 ± 0.04). This reduction may be attributed to the influence of KOH concentration, which has been identified as a key factor affecting carrageenan viscosity [11,22]. Lower viscosity is often associated with partial depolymerization of polysaccharide chains during alkaline treatment, highlighting the importance of carefully controlled extraction parameters in maintaining physicochemical integrity.

#### 3.2.4. ATF-FTIR Analysis

**Figure 3:**
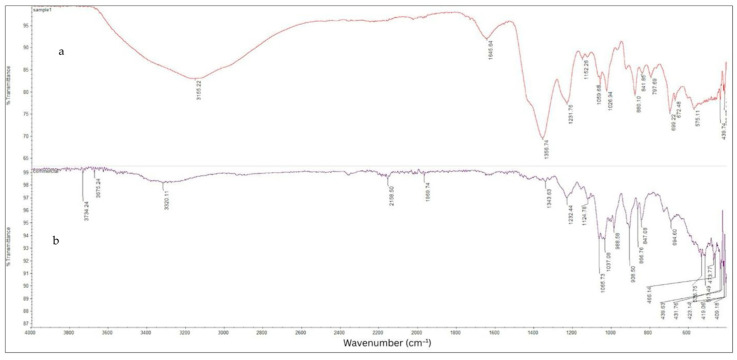
Infrared spectra of the crude carrageenan fraction from *E. perplexum* (**a**) and commercial carrageenan from (Sigma-Aldrich Co., St. Louis, MO, USA) product (**b**).

ATR-FTIR spectroscopy was employed to assess the impact of extraction conditions on the molecular structure and functional groups of carrageenan derived from *E. perplexum*. The spectral profiles of the extracted samples (Figure 3a) closely resembled those of the commercial standard (Figure 3b), with characteristic absorption bands within the 4000–400 cm^−1^ range, consistent with previous literature [17,49,51].

Prominent bands at 1026.94, 1037.08, 1059.68, and 1065.73 cm^−1^ were assigned to glycosidic linkages, falling within the typical 1010–1080 cm^−1^ range observed for carrageenan. O–H stretching vibrations were detected at 3155.22 and 3320.11 cm^−1^, closely matching the 3350 cm^−1^ band reported by Fan et al. [17]. A peak at 1645.64 cm^−1^ was attributed to water deformation, comparable to the 1637 cm^−1^ signal described by Mobarak et al. [52].

Absorption bands at 1231.76 and 1232.44 cm^−1^ corresponded to symmetric O=S=O and –O–SO_3_ stretching vibrations associated with D-galactose-4-sulfate (G4S), corroborating findings by Freile-Pelegrín et al. [53] and Tranquilan-Aranilla et al. [54]. Peaks at 841.85 and 847.08 cm^−1^ were indicative of G4S unit’s characteristic of κ- and ι-carrageenan, respectively [52,53], while a distinct band at 797.69 cm^−1^ confirmed the presence of D-galactose-2-sulfate (DA2S), a structural marker of ι-carrageenan [11,51,52]. Additionally, a signal at 1152.26 cm^−1^ was observed, corresponding to C–O bridge stretching vibrations, closely matching the 1156 cm^−1^ band reported by Jumaah et al. [51]. The consistent presence and positioning of these spectral features across all treatments suggest that variations in KOH concentration, extraction temperature, duration, and seaweed-to-water ratio did not significantly alter the core chemical structure of the extracted carrageenan.

## 4. Conclusions

This study demonstrated the utility of RSM to optimize extraction parameters for carrageenan from *E. perplexum*, resulting in improved yield and quality. The crude extract exhibited structural and functional attributes comparable to commercial κ-carrageenan, including favorable rheological behavior and superior emulsifying capacity, underscoring its potential for industrial applications. However, the scope of this work was limited to the characterization of crude carrageenan, without extensive evaluation of its bioactive potential. Future investigations should focus on purification, detailed structural analyses, and assessing its biological activities to fully elucidate its functional and therapeutic applications.

## Figures and Tables

**Table 1:** Independent variables and corresponding coded and actual value levels in the optimization of carrageenan extraction from *E. perplexum*.

Independent Variable	Symbol	Coded Levels				
		−α	−1	0	1	+α
Extraction temperature (°C)	X1	59.77	70	85	100	110.23
Extraction time (h)	X2	1.32	2	3	4	4.68
KOH (M)	X3	0.28	0.89	1.78	2.67	3.28

**Table 2:** CCD experimental design with coded and actual levels and measured responses for carrageenan extraction from *E. perplexum*.

Runs	Independent Variables			Response Values (Y)
	Temperature, °C	Time, h	KOH, M	Yield, %
1	100	2	2.67	66.87
2	70	4	2.67	50.03
3	110.22	3	1.78	-*
4	85	4.68	1.78	74.40
5	85	3	1.78	65.20
6	85	3	3.28	77.49
7	85	1.32	1.78	61.94
8	100	2	0.89	0.00
9	100	4	0.89	0.00
10	85	3	1.78	60.64
11	85	3	1.78	72.32
12	85	3	1.78	61.87
13	85	3	1.78	66.02
14	100	4	2.67	64.31
15	85	3	1.78	60.86
16	70	2	2.67	60.67
17	85	3	0.28	0.00
18	70	4	0.89	0
19	70	2	0.89	0
20	59.77	3	1.78	66.03

The response (Y) was the mean value of three replicates. -*: not test.

**Table 3:** Analysis of variance (ANOVA) for response surface reduced quadratic model.

Source	Sum of Squares	df	Mean Square	F-Value	*p*-Value	
Model	363.74	4	90.94	3.72	0.0419	significant
B-Time	0.3832	1	0.3832	0.0157	0.9029	
C-KOH Concentration	77.41	1	77.41	3.17	0.1055	
BC	114.96	1	114.96	4.70	0.0553	
C^2^	241.26	1	241.26	9.87	0.0105	
Residual	244.49	10	24.45			
Lack of Fit	145.47	5	29.09	1.47	0.3416	not significant
Pure Error	99.02	5	19.80			
Cor Total	608.23	14				

**Table 4:** Color Properties for carrageenan extraction from *E. perplexum*.

Runs	Parameters			
	L*	a*	b*	Total Color Difference (Δ*E*)
1	94.90 ± 0.91 ^a^	−11.81 ± 1.06 ^a^	36.11 ± 6.74 ^c^	10.86 ± 6.74 ^ab^
2	93.44 ± 1.10 ^ab^	−12.17 ± 0.80 ^abc^	41.44 ± 2.35 ^bc^	5.41 ± 2.40 ^abcd^
3	94.67 ± 1.07 ^a^	−11.93 ± 0.59 ^ab^	37.05 ± 6.06 ^c^	9.90 ± 6.06 ^abc^
4	90.88 ± 1.90 ^abc^	−13.88 ± 0.31 ^def^	46.54 ± 0.53 ^ab^	2.41 ± 0.89 ^cd^
5	90.67 ± 0.80 ^abc^	−14.60 ± 0.41 ^ef^	48.81 ± 1.17 ^ab^	3.72 ± 0.82 ^bcd^
6	91.72 ± 3.29 ^ab^	−12.17 ± 0.38 ^abc^	46.42 ± 2.96 ^ab^	0.00 ± 0.00 ^d^ (Control)
7	88.84 ± 5.85 ^abc^	−13.51 ± 0.68 ^cdef^	48.36 ± 2.23 ^ab^	5.15 ± 4.56 ^abcd^
10	84.70 ± 3.44^c^	−14.50 ± 1.18 ^ef^	47.17 ± 1.14 ^ab^	7.64 ± 3.19 ^abcd^
11	88.58 ± 4.27 ^abc^	−14.77 ± 0.55 ^ef^	46.91 ± 1.69 ^ab^	5.23 ± 2.47 ^abcd^
12	87.57 ± 3.27 ^bc^	−14.27 ± 0.57 ^ef^	48.97 ± 0.87 ^ab^	5.57 ± 2.79 ^abcd^
13	84.91 ± 5.90 ^c^	−13.42 ± 0.81 ^bcde^	50.18 ± 0.76 ^a^	8.53 ± 4.49 ^abc^
14	94.27 ± 3.82 ^ab^	−11.24 ± 1.37 ^a^	36.75 ± 11.80 ^c^	12.35 ± 8.86 ^a^
15	88.73 ± 4.32 ^abc^	−15.04 ± 0.33 ^f^	46.20 ± 1.90 ^ab^	5.35 ± 2.46 ^abcd^
16	87.53 ± 3.95 ^bc^	−12.45 ± 1.10 ^abcd^	51.47 ± 1.25 ^a^	7.35 ± 1.72 ^abcd^
20	91.14 ± 1.98 ^abc^	−14.50 ± 0.99 ^ef^	48.43 ± 1.95 ^ab^	3.68 ± 1.80 ^bcd^

L* value indicates lightness; a* indicates redness; b* indicates yellowness. Values are expressed as mean ± standard deviation (*n* = 3). Within each column, means that share the same superscript letter are not significantly different (*p* > 0.05), while those with different letters indicate statistically significant differences (*p* < 0.05).

## Data Availability

The original contributions presented in this study are included in the article; further inquiries can be directed to the corresponding authors.
